# Role of Necrosectomy in Necrotizing Pancreatitis: A Narrative Review

**DOI:** 10.7759/cureus.70470

**Published:** 2024-09-29

**Authors:** Khushbu Vaidya, Raju K Shinde, Tushar Nagtode, Ashish Jivani, Somya Goel, Joben Samuel

**Affiliations:** 1 General Surgery, Jawaharlal Nehru Medical College, Datta Meghe Institute of Higher Education and Research, Wardha, IND; 2 General Surgery, Jawaharlal Nehru Medical College, Datta Meghe Institute of Higher Education and Researc, Wardha, IND

**Keywords:** minimally invasive surgery, necrosectomy, necrotising pancreatitis, pancreatic necrosis, step-up approach

## Abstract

Necrotizing pancreatitis (NP) is a severe complication of acute pancreatitis, characterized by necrosis of pancreatic and peripancreatic tissues, leading to significant morbidity and mortality. The role of necrosectomy, the surgical removal of necrotic tissue, in the management of NP has evolved over the past few decades, moving from early aggressive surgical intervention to a more conservative and stepwise approach. This narrative review explores the historical perspectives, current practices, and future trends in the role of necrosectomy in NP. Early studies favored open surgical debridement; however, high mortality rates associated with early intervention prompted a shift towards minimally invasive techniques, delayed interventions, and the “step-up approach,” combining percutaneous drainage with minimally invasive surgery. We also review the indications for surgery, optimal timing, and various techniques, including video-assisted retroperitoneal debridement and endoscopic transluminal necrosectomy. The review highlights the benefits of these strategies in reducing complications, improving patient outcomes, and minimizing hospital stays. Ongoing research into patient selection, timing, and procedural refinement will continue to shape the role of necrosectomy in NP management. Understanding the evolving role of necrosectomy is crucial for optimizing care and reducing the burden of this life-threatening condition.

## Introduction and background

Overview of necrotizing pancreatitis (NP)

NP is a severe form of acute pancreatitis characterized by inflammation and necrosis of pancreatic and peripancreatic tissue. It typically develops as a complication of acute pancreatitis, where enzyme autodigestion, tissue ischemia, and inflammation result in extensive pancreatic tissue necrosis. In contrast to interstitial pancreatitis, NP involves necrotic tissue that can become infected, significantly increasing morbidity and mortality rates. Approximately 10%-20% of patients with acute pancreatitis develop NP, often leading to severe systemic complications such as sepsis, multi-organ failure, and metabolic derangements [1]. The pathophysiology of NP is driven by a cascade of inflammatory responses, leading to pancreatic tissue destruction and systemic inflammatory response syndrome (SIRS) [2]. The clinical significance of NP lies in its potential to cause both local and systemic complications. Locally, NP may lead to the formation of walled-off necrosis (WON), pancreatic abscesses, or pseudocysts, while systemic effects include septic shock and multi-organ failure [3]. Infected pancreatic necrosis (IPN) is a critical complication, occurring in up to 40% of patients and often necessitating surgical intervention [4]. Despite advances in medical management, mortality in NP remains high, especially when infection and organ failure are present.

The rationale for surgical intervention

Given the significant risk of infection and other complications in NP, surgical intervention has emerged as a critical aspect of its management. Historically, open surgery was the primary approach for debridement of necrotic tissue. However, minimally invasive techniques, including endoscopic and percutaneous interventions, are now more commonly employed. Necrosectomy, the surgical removal of necrotic pancreatic tissue, plays a pivotal role in the management of NP, particularly in cases of infected necrosis [5]. While conservative management is preferred in sterile necrosis, necrosectomy is indicated when infection or persistent organ dysfunction occurs. Necrosectomy serves to control infection, limit systemic inflammatory responses, and prevent further complications such as hemorrhage or perforation of surrounding organs [6]. Recent advances in surgical techniques have improved patient outcomes by reducing mortality and morbidity associated with traditional open surgery. Nonetheless, the timing, method, and necessity of necrosectomy remain areas of active investigation. Current guidelines recommend a delayed approach, allowing for the maturation of necrotic collections and stabilization of the patient before intervention [7]. With the evolving landscape of surgical and endoscopic interventions, it is imperative to re-evaluate the role of necrosectomy in NP management. Minimally invasive techniques have shown promising outcomes, yet the decision to perform necrosectomy should be tailored to each patient's condition. Understanding the optimal timing, approach, and long-term outcomes of necrosectomy in NP is essential to improving patient care. This narrative review aims to provide an updated examination of the role of necrosectomy in NP, highlighting current practices, emerging trends, and future directions.

## Review

Search methodology

The search methodology for this narrative review involved a comprehensive literature search of several electronic databases, including PubMed, MEDLINE, Embase, and Google Scholar, from inception to September 2024. Relevant keywords and Medical Subject Headings (MeSH) terms such as “necrosectomy,” “necrotizing pancreatitis,” “surgical management,” “pancreatic necrosis,” and “minimally invasive surgery” were combined using Boolean operators (AND, OR) to maximize search sensitivity. Inclusion criteria included original studies, reviews, meta-analyses, and clinical guidelines published in English that discussed the role, indications, techniques, and outcomes of necrosectomy in NP. Articles focusing on both open and minimally invasive necrosectomy approaches were included. Studies limited to animal models or unrelated to the topic were excluded. The reference lists of relevant articles were manually searched to identify additional pertinent studies. Titles and abstracts were screened independently by two reviewers, followed by a full-text review to ensure relevance to the study objectives.

Historical perspective of necrosectomy in NP

Early Surgical Approaches to Managing NP

NP, a severe and life-threatening form of acute pancreatitis, has long been managed through surgical interventions aimed at removing necrotic tissue, a procedure known as necrosectomy. In the early 20th century, surgical management was aggressive, with early laparotomies and open necrosectomy being the predominant approaches to treating NP. These procedures often involved extensive removal of necrotic pancreatic and peripancreatic tissue. Open necrosectomy, characterized by its invasive nature, was traditionally performed to prevent infection and organ failure due to the release of inflammatory mediators from the necrotic tissue. However, these interventions were associated with high morbidity and mortality rates, with complications such as pancreatic fistulae, infections, and bleeding being common post-surgical issues [8].

Evolution of Necrosectomy Techniques Over the Years

Over time, understanding the pathophysiology of NP and the risks associated with early and aggressive surgery led to a shift in the timing and approach of necrosectomy. By the late 20th century, it became evident that delaying surgery until the necrosis was walled off or infected significantly improved outcomes. This change in surgical strategy marked the transition from early intervention to a more conservative, “step-up” approach, which prioritizes less invasive procedures initially [9]. The evolution of surgical techniques also saw the introduction of retroperitoneal approaches to reduce the invasiveness of necrosectomy. The development of minimally invasive surgery, such as video-assisted retroperitoneal debridement (VARD), emerged as a major advancement in the field. VARD allowed surgeons to access the necrotic tissue through a smaller incision, reducing the risks of complications associated with open surgery. Similarly, techniques such as endoscopic necrosectomy, which utilizes endoscopic tools to debride the necrotic areas through the gastrointestinal tract, became increasingly popular [5].

Outcomes and Complications in Early Practice

In the early days of managing NP, outcomes following open necrosectomy were often poor, with mortality rates reported as high as 50% in severe cases. This was largely due to the high risk of postoperative complications, such as infection, multi-organ failure, and pancreatic fistula formation. These complications extended hospital stays and increased the need for additional interventions [10]. The high rate of morbidity also led to frequent re-operations, with many patients requiring multiple surgeries to manage ongoing infections or incomplete debridement of necrotic tissue. In addition to surgical risks, the postoperative care of patients was often complicated by prolonged intensive care stays, the need for parenteral nutrition, and complex wound management [11].

Introduction of Less Invasive Techniques Over Time

The introduction of less invasive techniques revolutionized the management of NP. Percutaneous drainage, for example, was initially developed as a supportive measure to manage fluid collections and infection in NP. Over time, it became apparent that percutaneous drainage could sometimes prevent the need for surgical necrosectomy altogether, especially in cases where infection was adequately controlled. This led to the establishment of the “step-up” approach, where percutaneous drainage is performed first, followed by minimally invasive necrosectomy if necessary [12]. Endoscopic approaches, particularly endoscopic transgastric or transduodenal drainage, and debridement, further transformed the landscape of NP management. These techniques allowed for the drainage of pancreatic fluid collections and the removal of necrotic debris without needing an external incision. The advantages of such approaches include reduced recovery times, lower infection rates, and fewer complications compared to traditional open surgery [13]. Over the past two decades, randomized controlled trials and large cohort studies have consistently demonstrated the superiority of minimally invasive approaches over open necrosectomy in terms of patient outcomes and cost-effectiveness. As a result, these techniques have become the standard of care for managing NP, with open necrosectomy now being reserved for only the most severe or refractory cases [14].

Current indications for necrosectomy

Clinical Symptoms and Timing of Intervention

Infected necrosis: The most common and well-established indication for necrosectomy is infected pancreatic necrosis. Clinical signs of sepsis, including fever, leukocytosis, and positive cultures from percutaneous aspiration, typically confirm infected necrosis. These findings often necessitate intervention due to the increased risk of systemic complications and death if left untreated. Delayed intervention is generally preferred to allow differentiation of necrotic tissue, which can reduce procedural complications and improve outcomes [15].

Persistent organ failure: Another critical indication for necrosectomy is persistent organ failure, particularly in patients experiencing multi-organ dysfunction (MODS) that does not resolve with non-surgical interventions. Organ failure in NP is a marker of disease severity, and prolonged organ dysfunction may suggest that surgical intervention is required to remove necrotic tissue, contributing to the ongoing systemic inflammatory response [5].

Symptomatic sterile necrosis: While sterile pancreatic necrosis is typically managed conservatively, there are cases where patients with extensive sterile necrosis may develop persistent symptoms, such as unrelenting abdominal pain, gastric outlet obstruction, or biliary obstruction. These symptoms are often due to the mechanical effects of large necrotic collections or inflammation, and in such cases, necrosectomy may be warranted despite the lack of infection [16].

Radiological Signs Indicating Necrosis

Presence of WON: WON represents a mature stage of NP, where necrotic tissue becomes encapsulated by a well-defined fibrous wall. This process typically occurs about four weeks after the onset of necrosis. The encapsulation helps demarcate the necrotic tissue from the surrounding viable tissue, making it easier to identify and target during debridement procedures. WON is typically detected using contrast-enhanced computed tomography (CECT) or magnetic resonance imaging (MRI), appearing as a heterogeneous collection with both liquid and solid components. The presence of WON is considered an important criterion for proceeding with necrosectomy, as the encapsulation indicates that the necrotic tissue is ready for safe and effective removal [1].

Gas bubbles in necrotic tissue: The presence of gas bubbles within necrotic pancreatic or peripancreatic collections, as visualized on CECT, is a strong indicator of infection. Gas within these collections usually results from gas-forming bacteria and signifies an infected necrotic collection associated with a high risk of sepsis and other life-threatening complications. Infected necrosis with gas bubbles often requires urgent intervention, including drainage and potential necrosectomy, as conservative management is rarely sufficient in such cases. Identifying gas within necrotic collections thus serves as a key radiological indication for necrosectomy [17].

Persistent fluid collections: Fluid collections that persist or enlarge over time, especially those that exert pressure on surrounding structures or cause symptoms, are another important radiological sign that may necessitate necrosectomy. These collections are often identified through serial imaging and may be associated with compressive symptoms such as gastric outlet obstruction, biliary obstruction, or abdominal pain. If persistent fluid collections are not amenable to percutaneous or endoscopic drainage, necrosectomy may be required to alleviate symptoms and prevent further complications. Imaging modalities like CECT and MRI are essential in tracking the progression of these collections, helping clinicians determine the need for surgical or endoscopic intervention [18].

Non-surgical Management Alternatives

Antibiotics: Prophylactic antibiotics are not routinely recommended for sterile necrosis in NP, as studies have shown no significant benefit in preventing infections. However, targeted antibiotic therapy becomes crucial in cases where there is a suspected or confirmed infection of the necrotic tissue. Common pathogens infecting necrotic pancreatic tissue include gram-negative bacteria, gram-positive cocci, and sometimes fungi. Broad-spectrum antibiotics such as carbapenems, quinolones, or metronidazole may be initiated based on clinical suspicion. Still, therapy should be tailored to the specific organism cultured from blood or drainage samples. Early and accurate diagnosis of infection is critical to improving patient outcomes, as infected necrosis carries a high risk of mortality if untreated [11].

Percutaneous or endoscopic drainage: Percutaneous or endoscopic drainage is often the first-line intervention in the “step-up” approach to managing infected or symptomatic walled-off pancreatic necrosis (WON). This technique involves the insertion of a catheter to drain fluid collections under imaging guidance (such as CT or ultrasound), which helps to relieve pressure, reduce infection risk, and control systemic inflammation. Endoscopic drainage, performed using endoscopic ultrasound (EUS), offers an alternative approach by allowing internal drainage into the gastrointestinal tract. These minimally invasive interventions often serve as a bridge to surgery, and in some cases, they can delay or completely avoid the need for surgical necrosectomy. Studies have shown that when these procedures are performed promptly and appropriately, the need for open surgery is reduced, improving overall morbidity and mortality outcomes [19].

**Figure 1:**
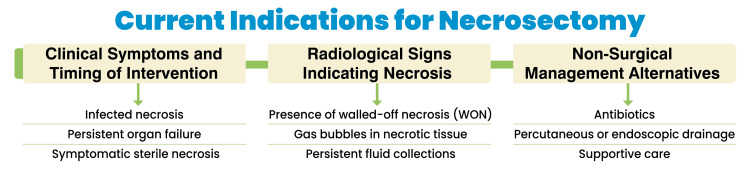
Current indications for necrosectomy Image credits: Dr. Khushbu Vaidya

Supportive care: Comprehensive supportive care is essential in managing NP, particularly in the acute phase of the disease. Intensive care support includes aggressive intravenous hydration to maintain circulatory stability and prevent organ failure, which is common in the early stages of the disease. Early nutritional support, ideally through enteral feeding, is preferred over parenteral nutrition, as it has been shown to reduce the risk of infection and promote gut integrity. Organ support, such as mechanical ventilation or renal replacement therapy, may be required for patients with multi-organ dysfunction. Careful monitoring of hemodynamics, electrolytes, and metabolic status is crucial, and timely escalation of care is essential in preventing complications [20]. Figure 1 shows current indications for necrosectomy.

Techniques of necrosectomy

Open Necrosectomy

Open surgery: Open necrosectomy has historically been the standard surgical treatment for NP, involving the removal of necrotic tissue through a large abdominal incision. This approach allows for direct visualization and manual debridement of necrotic tissue, offering thorough clearance. However, the procedure is associated with high morbidity and mortality rates, primarily due to the invasiveness and the risk of postoperative complications like infection, bleeding, and multiple organ failure [5].

Indications, advantages, and limitations: Open necrosectomy is typically reserved for patients with extensive necrosis, sepsis, or those who do not respond to less invasive treatments. The advantage of this approach lies in its capacity to address widespread necrosis in a single procedure. However, the limitations are significant, including a longer hospital stay, higher rates of postoperative complications, and prolonged recovery [21].

Minimally Invasive Techniques

Laparoscopic and endoscopic procedures: Minimally invasive techniques, such as laparoscopic and endoscopic necrosectomy, have gained prominence recently. These procedures aim to reduce the trauma associated with traditional open surgery. Laparoscopic techniques involve small incisions through which specialized instruments are introduced to debride necrotic tissue. At the same time, endoscopic necrosectomy is performed using an endoscope passed through the gastrointestinal tract to access the pancreas [22].

Step-up approach - percutaneous drainage to minimally invasive surgery: The step-up approach is a widely accepted method that begins with percutaneous drainage of pancreatic collections and progresses to minimally invasive surgery if necessary. Studies show this method effectively reduces the need for open surgery, as about one-third of patients can be successfully treated with drainage alone. If drainage is insufficient, a minimally invasive necrosectomy is performed to debride necrotic tissue [23].

Benefits of minimally invasive methods in reducing morbidity and mortality: Minimally invasive methods have been associated with reduced morbidity and mortality rates compared to open surgery. These techniques result in fewer complications, shorter hospital stays, and improved recovery outcomes. Furthermore, they are often performed in stages, allowing for more controlled interventions [23].

Video-Assisted Retroperitoneal Debridement

Overview and current usage: VARD is a hybrid technique that combines minimally invasive approaches with open surgery principles. It involves making a small incision in the flank to access the retroperitoneal space, followed by debridement of necrotic tissue under direct vision using video assistance. VARD is commonly used after the failure of percutaneous drainage in the step-up approach [14].

**Figure 2:**
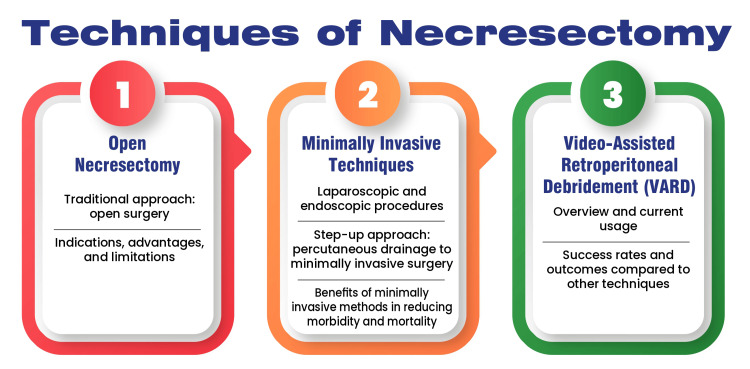
Techniques of necrosectomy Image credits: Dr. Khushbu Vaidya

Success rates and outcomes compared to other techniques: VARD has demonstrated higher success rates and better outcomes when compared to traditional open surgery and even some minimally invasive methods. Studies show that VARD offers a lower risk of organ failure and reduced mortality rates. Additionally, it has been noted to result in fewer postoperative complications, such as fistula formation and bleeding, compared to open necrosectomy [24]. Figure 2 shows techniques of necrosectomy.

Timing of necrosectomy

Early vs. Delayed Necrosectomy

Early necrosectomy: Traditionally, early necrosectomy was favored because it could prevent the progression of infection and systemic complications. However, recent evidence suggests that early surgical intervention may increase the risk of complications, particularly in the initial phase of the disease when the inflammatory process is still evolving. For instance, a study by Mier et al. found that early necrosectomy, defined as within the first three weeks of onset, was associated with increased surgical complications and higher mortality than delayed intervention [25].

Delayed necrosectomy: In contrast, delayed necrosectomy, performed after the initial inflammatory response has subsided (typically after three to four weeks), allows for a more accurate assessment of the extent of necrosis and minimizes the risk of harming viable pancreatic tissue. A seminal study highlighted that delayed necrosectomy improved overall survival rates and reduced perioperative morbidity [26]. The study showed that performing surgery after developing well-defined necrotic collections provided a more favorable risk-benefit ratio, leading to better patient outcomes.

Impact of Timing on Patient Outcomes and Mortality Rates

The impact of timing on patient outcomes and mortality rates has been the subject of extensive research. Evidence suggests that the timing of necrosectomy can significantly influence both survival and complication rates. For example, an article compared early and delayed necrosectomy in NP and found that delayed necrosectomy was associated with lower mortality rates and reduced risk of major complications, such as post-operative pancreatic fistula and wound infections [16]. The study emphasized that early surgery, before the necrotic tissue had sufficiently delineated, often led to higher rates of reoperation and sepsis. Another important study reinforced these findings, demonstrating that delayed intervention improved survival rates and enhanced the quality-of-life post-surgery [27]. The authors attributed this to the reduced inflammatory response and improved surgical outcomes associated with a more considered timing of the intervention.

Optimal Window for Surgical Intervention in NP

Determining the optimal window for necrosectomy in NP involves balancing the risks of early intervention with the benefits of timely surgical intervention. The consensus in recent literature leans towards a delayed approach, with most guidelines suggesting intervention after three to four weeks from the onset of symptoms. The International Consensus Guidelines advocate for a strategy of initial conservative management followed by surgical intervention once the patient has stabilized, and the necrotic tissue has become more clearly defined [28]. This approach minimizes unnecessary complications and improves the chances of a successful outcome. A recent review also supports this delayed approach, noting that the optimal timing for necrosectomy is highly patient-specific but generally falls within the window of three to four weeks from the onset of symptoms. This allows for better patient selection and surgical planning, ultimately improving outcomes [1].

Outcomes of necrosectomy

Morbidity and Mortality Associated With Necrosectomy

Morbidity: The rate of post-operative complications such as infections, bleeding, and organ dysfunction is high. According to a meta-analysis, the overall morbidity rate following necrosectomy ranges from 40% to 60% [29].

Mortality: The mortality rate associated with necrosectomy has been reported to be between 10% and 30%, largely dependent on the patient’s overall condition, the extent of necrosis, and the timing of the surgery [1].

Long-Term Survival and Quality of Life Following Surgery

Survival: Long-term survival following necrosectomy is closely linked to the timing and efficacy of the procedure. When performed promptly and effectively, necrosectomy can significantly improve survival outcomes in patients with NP. Studies have shown that early intervention can prevent the progression of systemic inflammation and sepsis, reducing the risk of multi-organ failure, a leading cause of mortality in severe cases. A notable study reported a five-year survival rate of approximately 70% for patients who underwent necrosectomy, demonstrating that, despite the complexity of the condition, survival prospects can be favorable with appropriate surgical management. However, survival rates may vary depending on factors such as the extent of pancreatic necrosis, the presence of co-morbidities, and post-operative complications such as infection or delayed gastric emptying. Additionally, patients who experience complications related to the underlying pancreatic disease may have lower survival rates [27].

Quality of life: While necrosectomy can enhance survival, many survivors face a substantial reduction in quality of life due to the long-term sequelae of NP and the surgical procedure itself. Chronic complications, including persistent abdominal pain, pancreatic insufficiency, and the development of diabetes mellitus due to pancreatic dysfunction, are common among survivors. These conditions often require ongoing medical management and significantly impact day-to-day functioning [30].

Post-operative Complications

Infections: Intra-abdominal infections, particularly pancreatic abscesses and sepsis are among the most prevalent complications following necrosectomy. These infections arise from the bacterial contamination of necrotic tissue and can significantly impact post-operative recovery. Patients who undergo necrosectomy are at high risk due to the invasive nature of the surgery and the already compromised state of the pancreas. Retrospective analysis indicated infection rates as high as 50% post-necrosectomy, which further prolonged hospital stays and increased the need for additional interventions, such as drainage or even re-operationally, these infections may escalate into systemic sepsis, a life-threatening condition, leading to multi-organ failure and increased mortality if not promptly managed [31].

Bleeding: Hemorrhage is another major complication, often resulting from the manipulation of vascular structures within and surrounding the pancreas during necrosectomy. The process of debriding necrotic tissue can inadvertently damage blood vessels, especially when the inflammation and necrosis have distorted the usual anatomy. Hemorrhage can occur intraoperatively or postoperatively, sometimes requiring urgent intervention. Approximately 15% of patients experience significant bleeding after necrosectomy, with some requiring blood transfusions, angiographic embolization, or even repeat surgery to control the hemorrhage. Postoperative bleeding can lead to further complications, such as anemia or hypovolemic shock, which can severely impact patient outcomes [32].

Pancreatic fistulas: Pancreatic fistulas are a common complication following the removal of necrotic pancreatic tissue. These fistulas form when pancreatic enzymes leak from damaged pancreatic ducts into surrounding tissues, creating an abnormal communication between the pancreatic system and other tissues or organs. The incidence of pancreatic fistulas post-necrosectomy is reported to range from 10% to 20%. These fistulas can lead to further morbidity, including persistent drainage, infection, electrolyte imbalances, and the need for prolonged hospital stays. In some cases, management of pancreatic fistulas requires additional interventions such as percutaneous drainage or endoscopic stenting, and in severe cases, repeat surgery. The long-term effects can also include pancreatic exocrine or endocrine insufficiency, which may require lifelong management [33].

Impact of Necrosectomy on Pancreatic Function

Exocrine function: Necrosectomy, the surgical removal of necrotic pancreatic tissue, can have a significant impact on pancreatic exocrine function. The pancreas is responsible for producing digestive enzymes, such as amylase, lipase, and proteases, which are critical for breaking down carbohydrates, fats, and proteins. When pancreatic tissue is removed, especially in large amounts, the production of these enzymes is reduced, leading to pancreatic exocrine insufficiency (PEI) [34].

Endocrine function: The pancreas also plays a vital role in endocrine regulation through the production of insulin, glucagon, and other hormones involved in glucose homeostasis. Necrosectomy can damage or remove insulin-producing beta cells in the islets of Langerhans, which may result in new-onset diabetes mellitus (NODM). The incidence of NODM following necrosectomy varies but is estimated to occur in 20% to 40% of patients' post-surgery [35].

Necrosectomy vs. conservative management

Surgical Management Outcomes

Necrosectomy has been associated with improved outcomes in specific contexts, especially when performed after the initial inflammatory phase has subsided. Early surgical intervention, however, may increase complications. Key studies indicate that delayed surgical necrosectomy (usually performed after four to six weeks) often results in better outcomes compared to early intervention [36]. It is essential to carefully time the surgery to avoid unnecessary complications while effectively managing necrotic tissue.

Conservative Management Outcomes

Conservative management focuses on intensive supportive care, including fluid resuscitation, pain management, and nutritional support. This approach is often preferred in the initial stages of NP, with surgical intervention reserved for cases that do not improve with medical management [37]. Conservative management has been shown to be effective in reducing the need for early surgery and minimizing the associated risks [38].

Future directions and innovations in necrosectomy

Emerging Techniques and Technologies

Robotics: The use of robotic-assisted surgery in necrosectomy represents a significant advancement. Robotic systems, such as the da Vinci Surgical System, offer enhanced precision, reduced tremor, and improved dexterity compared to traditional laparoscopic techniques. Studies suggest that robotic-assisted necrosectomy can lead to less postoperative pain, shorter recovery times, and potentially fewer complications [39]. Future research should focus on optimizing robotic techniques for complex cases and comparing them directly with conventional approaches in large-scale studies.

Advanced imaging: Advanced imaging technologies, including high-resolution computed tomography (CT), MRI, and EUS, are critical for preoperative assessment and intraoperative navigation during necrosectomy. Innovations in imaging, such as functional MRI and real-time intraoperative imaging, can improve the accuracy of necrotic tissue identification and delineation [40]. Continued development in imaging modalities and integration with surgical navigation systems will enhance the precision of necrosectomy.

Hybrid procedures: Hybrid procedures that combine endoscopic and surgical techniques are gaining attention in the management of NP. These approaches can minimize the invasiveness of surgery while allowing for comprehensive necrosectomy. For example, endoscopic-guided percutaneous necrosectomy (EPN) followed by laparoscopic debridement can offer a less invasive alternative to traditional open surgery [41]. Future research should evaluate the efficacy, safety, and long-term outcomes of hybrid approaches compared to standard surgical methods.

Research Gaps and Potential Areas for Future Exploration

Standardization of protocols: Despite advancements, there is a lack of standardized protocols for necrosectomy, particularly regarding the timing and criteria for intervention. Research should focus on developing and validating guidelines to standardize the management of NP, including the timing of necrosectomy and the use of adjunctive therapies [42].

Long-term outcomes and quality of life: Long-term outcomes following necrosectomy, including survival rates, recurrence of pancreatic necrosis, and quality of life, remain underexplored. Comprehensive longitudinal studies are needed to assess the long-term impacts of various surgical strategies and to identify factors that predict better outcomes [43].

Personalized medicine: Personalized approaches to managing NP are an emerging area of interest. Understanding the genetic, molecular, and immunological factors influencing disease progression and response to treatment can lead to more tailored therapeutic strategies. Future research should explore the role of biomarkers and genetic profiling in predicting outcomes and customizing treatment plans [44].

Personalized Approaches to Managing NP

Personalized medicine in NP involves tailoring treatment strategies based on individual patient characteristics, including genetic, molecular, and clinical factors. Advances in genomics and proteomics can provide insights into patient-specific disease mechanisms and treatment responses. For instance, identifying specific genetic mutations or biomarkers associated with severe NP could guide more precise and effective management strategies [45]. Additionally, integrating personalized medicine approaches with emerging technologies, such as targeted drug delivery systems and individualized surgical techniques, holds promise for improving patient outcomes. Future research should focus on validating these personalized approaches through clinical trials and integrating them into routine clinical practice.

## Conclusions

In conclusion, necrosectomy plays a crucial role in the management of NP, particularly in cases where infected pancreatic necrosis or persistent organ failure develops. The evolution of surgical techniques from open necrosectomy to minimally invasive approaches, such as VARD and endoscopic transluminal necrosectomy, has significantly improved patient outcomes by reducing morbidity and mortality. Optimal timing of intervention, typically after the maturation of necrotic tissue, is critical to minimize complications. While necrosectomy remains a cornerstone in treating severe NP, a multidisciplinary approach involving delayed drainage, use of antibiotics, and supportive care continues to be essential in improving overall patient prognosis.
